# Temporal sampling and network analysis reveal rapid population turnover and dynamic migration pattern in overwintering regions of a cosmopolitan pest

**DOI:** 10.3389/fgene.2022.986724

**Published:** 2022-08-30

**Authors:** Fushi Ke, Jianyu Li, Liette Vasseur, Minsheng You, Shijun You

**Affiliations:** ^1^ State Key Laboratory of Ecological Pest Control for Fujian-Taiwan Crops, Institute of Applied Ecology, Fujian Agriculture and Forestry University, Fuzhou, China; ^2^ Joint International Research Laboratory of Ecological Pest Control, Ministry of Education, Fuzhou, China; ^3^ Ministerial and Provincial Joint Innovation Centre for Safety Production of Cross-Strait Crops, Fujian Agriculture and Forestry University, Fuzhou, China; ^4^ School of Biological Sciences, The University of Hong Kong, Pok Fu Lam, Hong Kong SAR, China; ^5^ Institute of Plant Protection, Fujian Academy of Agricultural Sciences, Fuzhou, China; ^6^ Department of Biological Sciences, Brock University, St. Catharines, ON, Canada; ^7^ BGI-Sanya, Sanya, China

**Keywords:** temporal sampling, kinship analysis, population network, dynamic metapopulation, insect pest

## Abstract

Genetic makeup of insect pest is informative for source-sink dynamics, spreading of insecticide resistant genes, and effective management. However, collecting samples from field populations without considering temporal resolution and calculating parameters related to historical gene flow may not capture contemporary genetic pattern and metapopulation dynamics of highly dispersive pests. *Plutella xylostella* (*L.*), the most widely distributed Lepidopteran pest that developed resistance to almost all current insecticides, migrates heterogeneously across space and time. To investigate its real-time genetic pattern and dynamics, we executed four samplings over two consecutive years across Southern China and Southeast Asia, and constructed population network based on contemporary gene flow. Across 48 populations, genetic structure analysis identified two differentiated insect swarms, of which the one with higher genetic variation was replaced by the other over time. We further inferred gene flow by estimation of kinship relationship and constructed migration network in each sampling time. Interestingly, we found mean migration distance at around 1,000 km. Such distance might have contributed to the formation of step-stone migration and migration circuit over large geographical scale. Probing network clustering across sampling times, we found a dynamic *P. xylostella* metapopulation with more active migration in spring than in winter, and identified a consistent pattern that some regions are sources (e.g., Yunnan in China, Myanmar and Vietnam) while several others are sinks (e.g., Guangdong and Fujian in China) over 2 years. Rapid turnover of insect swarms and highly dynamic metapopulation highlight the importance of temporal sampling and network analysis in investigation of source-sink relationships and thus effective pest management of *P. xylostella*, and other highly dispersive insect pests.

## Introduction

Insect pests are abundant in all agricultural regions of the world, and many are difficult to control due to their rapid development of insecticide resistance and active migration. For these pests, molecular evidence of genetic pattern and gene flow may provide important guidance in understanding the colonization history (Yang et al., 2012; Kirk et al., 2013; Cao et al., 2019; You et al., 2020), tracking insecticide resistance (Taylor et al., 2021), and investigating metapopulation dynamics (Dinsdale et al., 2012; Elameen et al., 2020; Perera et al., 2020). Much work has been done on the genetic structure and phylogeography of widely distributed insect pests, such as *Locusta migratoria* (Ma et al., 2012), *Frankliniella occidentalis* (Yang et al., 2012; Cao et al., 2019), *Nilaparvata lugens* (Stål) (Hu et al., 2019) and *Plutella xylostella* (You et al., 2020). However, little is known for contemporary dynamics of insect pests that migrate actively all year-round (Fu et al., 2014) and form migration circuit between source-sink populations (Gao et al., 2020). Analysis of real-time pattern of genetic variation and contemporary gene flow thus would help to clarify source-sink metapopulation dynamics and insecticide resistance development.

Unlike vertebrate migrants, migration circuits of insects can involve multiple generations and experience different levels of connectivity due to their shorter generation time and lower degree of control over movement (flight) directions (Gao et al., 2020). Most population genetic studies of highly dispersive species report a pattern of low genetic differentiation and high gene flow (Jiang et al., 2010; Wei et al., 2013; Elameen et al., 2020), which is not unexpected, but not informative. This is due to the frequently applied genetic parameter *N*_m_ refers to historical gene flow and thus inadequate for contemporary population dynamics. In addition, *Nm* calculated by *F*_ST_ need several assumptions that real populations are likely violating and do not provide accurate estimation of migration (Whitlock and Mccauley, 1999). To better understand the contemporary genetic pattern and migration dynamics, choosing the suitable spatial and temporal resolution as well as the right metrics thus is vital (Osborne and Woiwod, 2002).For insect species with partial migration (i.e., a population consisting of migrants and residents) (Menz et al., 2019), pedigree-based approaches can be useful in identifying contemporary migration events (Chen et al., 2021). Furthermore, quantifying contemporary gene flow between populations by counting kinship assignments (Wang, 2014) can lead to the construction of population networks and identification of source-sink relationships. Population network analysis that uses populations (sites) as nodes and genetic similarity (or gene flow) as edges (Dyer, 2015) could further identify key nodes that affect temporal connectivity of metapopulation.

*Plutella xylostella* (diamondback moth, DBM) has developed resistance to almost all insecticides, causing enormous damage to global cruciferous vegetable and oil crop industries (Furlong et al., 2013; Li et al., 2016). It has been recorded in over 140 countries and regions worldwide, including Svalbard Island near the north pole (Coulson et al., 2002) and subantarctic Marion Island (Chown and Crafford, 1987). DBM is one of the most successful moths according to Miyata’ classification (Miyata, 1983). It is characterized by having high migration ability and with large-scale distribution of host plants. Migration and colonization of DBM are regionally and temporally heterogeneous. For example, DBM can overwinter in tropic and subtropic regions, but can only breed at particular time intervals at higher latitudes, where it must recolonize every year under suitable weather conditions (Chen et al., 2021; Ma et al., 2021). This forms a dynamic metapopulation (Ke et al., 2019) between overwintering habitats (e.g., lower latitude) and seasonally breeding regions (e.g., higher latitude). In northern Europe, warm currents from western Russia facilitate colonization of DBM at high-latitude area close to the north pole, while its presence in Finland is due to northward wind from Estonia (Tiilikkala et al., 1996). In England, winds from the mainland Europe bring DBM seasonally, with individuals of the early summer coming from East and Southeast Europe, and those arriving in late summer are from the eastern regions of Baltic Sea (Chapman et al., 2002). In Eastern Asia, mass migration of DBM is known to cross the Bohai Strait, with population density varying with the season and peaking in June and August (Fu et al., 2014). Monsoons (Ke et al., 2015) and typhoons (Kohno et al., 2004) also aid the migration of DBM across the open seas, resulting in their colonization of islands far from the continent. Dosdall et al. (2001) suggest that continuously strong winds in North America bring DBM from Southern United States and Mexico into Canada. Overall, spatially and temporally varied migration of DBM is influenced by external factors that formed the “weather window,” including wind strength and direction (Coulson et al., 2002).

East Asia is one of the most important regions for mass insect migration. Every year, a large number of insects crossing the sea (e.g., Bohai gulf and South China Sea) are recorded by radar (Guo et al., 2020; Zhou et al., 2021). Among them, *P. xylostella* is an important agricultural pest that migrate temporally and regionally (Fu et al., 2014; Wang et al., 2022). Widely distributed host plants (e.g., cultivated cruciferous vegetables) and suitable weather make East Asia an ideal place for DBM’s reproduction and migration throughout the year (Fu et al., 2014; Li et al., 2016). In this study, we hypothesized that temporal variation of DBM population dynamics has contributed to the differentiation of contemporary genetic pattern, source-sink relationships, and regional population clustering. To test this hypothesis, we conducted longitudinal sampling of *P. xylostella* populations in Southern China and Southeastern Asia during two consecutive years and investigated contemporary genetic makeup of these populations. We first probed temporal variation of genetic pattern in overwintering regions of DBM populations. Further, we applied kinship-based inference of gene flow to investigate contemporary metapopulation dynamics. Finally, by constructing population network of temporally sampled populations in each sampling time interval, we identified key populations that were important in regional dynamics. Our study represented the first documentation that employed temporal sampling and network analysis based on contemporary gene flow.

## Materials and methods

### Sampling design

**FIGURE 1:**
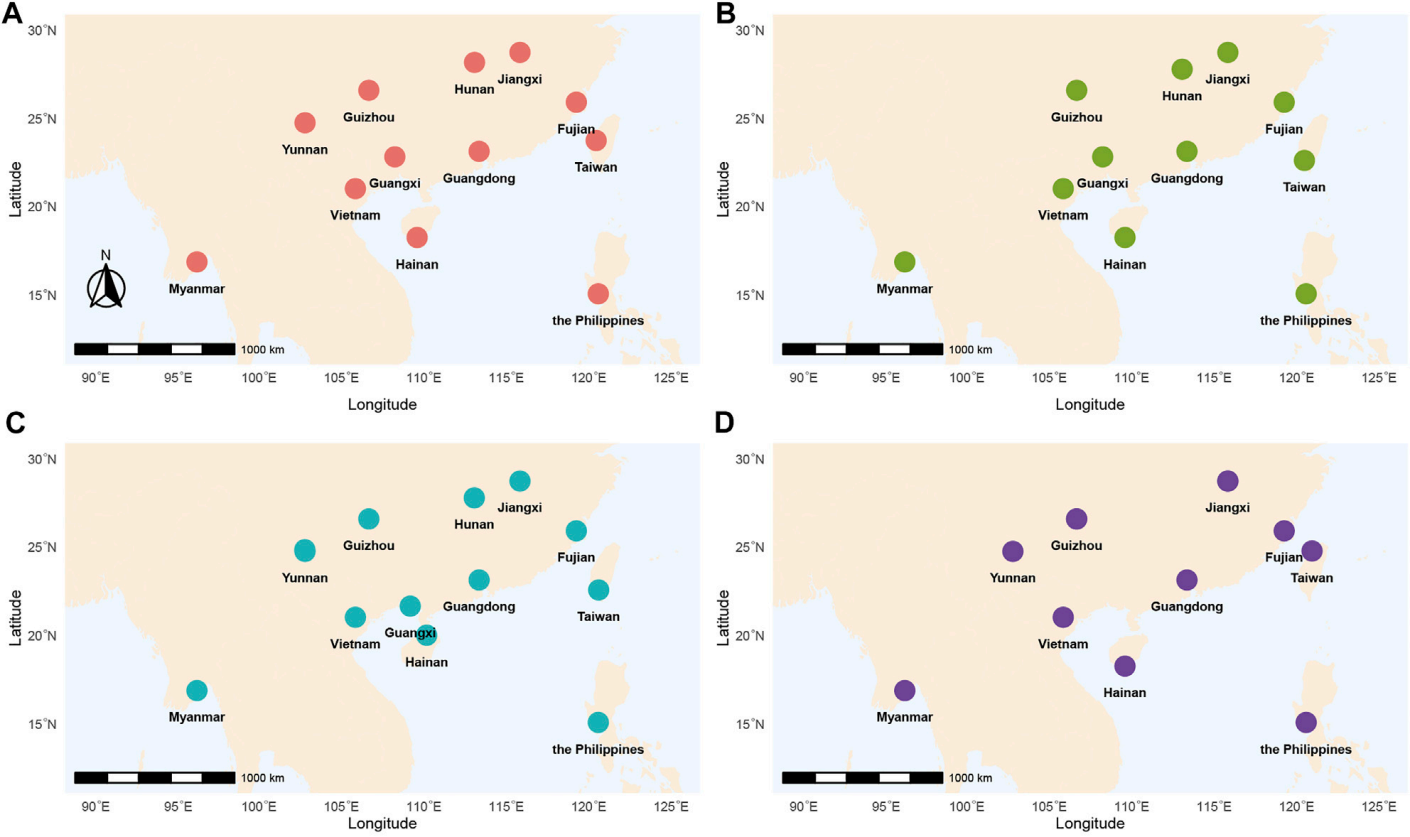
Geographical locations of *P. xylostella* sampling sites across Southern China and Southeast Asia. Different colors represented time1 (from April to June 2016) **(A)**, time 2 (from October 2016 to January 2017) **(B)**, time 3 (from April to June 2017) **(C)**, time 4 (from November 2017 to January 2018) **(D)**, respectively. Refer to Supplementary Table S1 for detailed information of collecting samples.

We implemented a longitudinal sampling of *P. xylostella* populations in four Asian countries including China, Myanmar, the Philippines, and Vietnam (Figure 1; Table 1). Specifically, one site from each of the nine provinces in China (Fujian, Guangdong, Guangxi, Guizhou, Hainan, Hunan, Jiangxi, Taiwan, Yunnan), and three additional sites from Myanmar, the Philippines, and Vietnam were selected and sampled twice a year (first sampling: April, May and June; second sampling: December and January) from April 2016 to January 2018. Note that three sites failed to be sampled at one specific sampling time (Yunnan in time 2, Hunan in time 4 and Guangxi in time 4, Figure 1; Table 1). In each site, we collected individuals at a distance of at least 3 m (to avoid sampling too many individuals with close kinship) and stored them in 95% alcohol. The samples were further stored at −80°C before being used for DNA extraction. Without any congeneric species of DBM reported or identified in this region (You et al., 2020), we verified the species identity of all sampled individuals using morphological characteristics following You & Wei (You, 2007) instead of sequencing mitochondrial *COI* of every individual (You et al., 2020).

**TABLE 1:** Sampling information of *P. xylostella* populations across Southern China and Southeast Asia.

Sampling time code	Population	Sampling site	Latitude	Longitude	Sample size	Sampling date
time1	FJ1	Fujian	25.94392	119.25313	32	2016/5/12
	FL1	the Philippines	15.10136	120.58555	32	2016/6/21
	GD1	Guangdong	23.16017	113.35634	31	2016/6/16
	GX1	Guangxi	22.85021	108.2448	32	2016/6/3
	GZ1	Guizhou	26.61723	106.66834	32	2016/6/28
	HN1	Hainan	18.29119	109.59779	32	2016/4/28
	JX1	Jiangxi	28.76363	115.83489	32	2016/4/24
	MY1	Myanmar	16.90561	96.24384	32	2016/7/9
	TW1	Taiwan	23.77326	120.45462	32	2016/6/10
	VN1	Vietnam	21.04872	105.85288	29	2016/7/7
	XX1	Hunan	28.1974	113.07853	25	2016/7/7
	YN1	Yunan	24.78023	102.79866	32	2016/6/25
time2	FJ2	Fujian	25.94392	119.25313	20	2016/12/6
	FL2	the Philippines	15.10136	120.58555	32	2017/1/16
	GD2	Guangdong	23.16017	113.35634	32	2017/1/9
	GX2	Guangxi	22.85021	108.2448	32	2017/12/18
	GZ2	Guizhou	26.61723	106.66834	32	2016/12/23
	HN2	Hainan	18.29119	109.59779	32	2017/1/6
	JX2	Jiangxi	28.76363	115.83489	29	2016/10/25
	MY2	Myanmar	16.90561	96.24384	32	2016/12/22
	TW2	Taiwan	22.64479	120.4785	29	2016/10/22
	VN2	Vietnam	21.04872	105.85288	32	2016/12/18
	XX2	Hunan	27.81154	113.06472	32	2016/12/20
time3	FJ3	Fujian	25.94392	119.25313	32	2017/4/27
	FL3	the Philippines	15.10136	120.58555	32	2017/5/6
	GD3	Guangdong	23.16017	113.35634	32	2017/5/23
	GX3	Guangxi	21.68073	109.17835	32	2017/4/29
	GZ3	Guizhou	26.61723	106.66834	32	2017/6/6
	HN3	Hainan	20.03826	110.17471	5	2017/5/15
	JX3	Jiangxi	28.76363	115.83489	32	2017/5/17
	MY3	Myanmar	16.90561	96.24384	32	2017/6/4
	TW3	Taiwan	22.59524	120.60754	32	2017/5/24
	VN3	Vietnam	21.04872	105.85288	21	2017/6/1
	XX3	Hunan	27.81154	113.06472	32	2017/5/25
	YN3	Yunnan	24.78023	102.79866	32	2017/6/5
	YC3	Yunan	24.87667	102.78666	14	2017/5/22
time4	FJ4	Fujian	25.94392	119.25313	32	2017/11/7
	FL4	the Philippines	15.10136	120.58555	32	2017/11/20
	GD4	Guangdong	23.16017	113.35634	32	2017/11/25
	GB4	Guizhou	26.61831	106.66114	32	2017/12/2
	GL4	Guizhou	26.61723	106.66834	32	2017/12/1
	HN4	Hainan	18.29119	109.59779	32	2018/1/26
	JX4	Jiangxi	28.76363	115.83489	32	2017/12/15
	JX5	Jiangxi	28.76363	115.83489	6	2018/1/6
	MY4	Myanmar	16.90561	96.24384	32	2017/11/28
	TW4	Taiwan	24.80418	120.94269	32	2018/1/26
	VN4	Vietnam	21.04872	105.85288	32	2017/11/26
	YN4	Yunan	24.78023	102.79866	32	2017/11/29

### DNA extraction and microsatellite genotyping

We extracted total genome DNA individually by using Gentra Puregene Blood Kit (QIAGEN) following the instruction manual. The extracted DNA of each individual was tested based on OD 260/280 ratio measured by UV-1600 Spectrophotometer. All the individuals were with high DNA concentration and quality, and suitable for microsatellite (i.e., simple sequence repeats, SSR) genotyping.

A total of 15 published SSR markers were selected for genotyping (Esselink et al., 2006; Ke et al., 2015). The PCR program with three primers based on Schuelke’ method (Schuelke, 2000) was employed. A more detailed description of the PCR reaction system can be found in our previous study (Ke et al., 2015). Briefly, the total volume of each PCR reaction was 25 μl, containing 12.5 μl Mix (Promega), 0.2 μl forward primer, 1 μl reverse primer, and 0.8 μl M-13 linked forward primer. The temperature conditions were set at 94°C for 10 min, and then 32 cycles at 94°C for 30 s, 56°C for 45 s, 72°C for 45 s, followed by eight cycles at 94°C for 30 s, 53°C for 45 s, 72°C for 45 s, and a final extension at 72°C for 10 min. PCR products were tested by gel electrophoresis, and reactions with no visible products were re-amplified with more PCR circles. All fluorescence-labelled PCR products were further scanned by ABI 3730 sequencer in Sangon Biotech (Shanghai). Sizes of the products were assigned by GeneMapper 4.1 (Applied Biosystems) and further checked manually.

### Genetic variation and structure

We used GenAIEx6.5 (Peakall and Smouse, 2006) and fastat (Goudet, 2001) to calculate several metrics of population genetic variation, including number of alleles (*Na*), effective population size (*Ne*), Shannon index (*I*), observed heterozygosity (*Ho*), expected heterozygosity (*He*), and allelic richness (*Ar*). In addition, we treated populations of the same sampling time interval as a metapopulation and estimated genetic variation in each metapopulation by using number of alleles and number of private alleles based on the rarefaction method in ADZE (Szpiech et al., 2008). We also performed the pricipla coordinates analysis (PCoA) based on calculation of Nei’s distance between populations in GenAIEx6.5 (Peakall and Smouse, 2006). The genetic structure of all sampled populations was further inferred using STRUCTURE (Pritchard et al., 2000) with 100,000 burn-in and 1,000,000 MCMC iterations. LOCPRIOR model was set with geographical information of populations (Hubisz et al., 2009). Each K from 1 to 5 was run with 10 replications, and the best K was determined by using STRUCTRE harvest (Earl and Vonholdt, 2012). The geographical distance of each sampling site pairs was measured by R package GEOSPHERE 1.5-10 (Fick and Hijmans, 2017) and used for regression analysis with Nei’s distance in GraphPad Prism 8 (Swift, 1997).

### Kinship assignment and contemporary gene flow analysis

Parentage inference based on individual genotypes of natural populations is important and has attracted research interests for decades (Jones et al., 2010). Genetic information of multiple offspring that share the same parent can provide important information for inferring parental genotype and is applied in joining sibship and parentage analysis based on likelihood (Wang and Santure, 2009) and Bayesian methods (Emery et al., 2001). Here, we used the Colony program (Jones and Wang, 2010) that could assign sibship and parentage jointly based on the full likelihood (FL) method. For populations in each sampling time interval, we conducted five long runs with settings including polygamy for both sexes, mating system with inbreeding, and high likelihood precision. To avoid bias due to small sampling size, populations with less than 10 individuals were excluded for this analysis. In addition, as the microsatellite loci can easily have genotyping errors, we further performed analysis based on different error rates (0.0001, 0.001, 0.01, 0.05) to check the marker informativeness (Wang, 2018). If these loci could provide essentially the same results under different error rates, then the markers were deemed informative and kinship assignment analysis accurate (Wang, 2018).

Based on the parentage relationships identified by Colony, we then investigated gene flow by calculating likelihood estimator of migration rates among the sampled populations in MigEst 1.0 (Wang, 2014). This software implements marker-based parentage assignments and jointly estimates contemporary migration rates (*m*) across the sampled populations from a metapopulation (Wang, 2014). Recent analysis indicates the coalescent-based method [e.g., Migrate-n (Beerli, 2006)] and disequilibrium estimates [e.g., BayesAss (Wilson and Rannala, 2002)] may lead to inaccurate estimates of gene flow under recent landscape change (Samarasin et al., 2017). Unlike BayesAss (Wilson and Rannala, 2002) that estimates recent gene flow in the last 5–15 generations and assumes low migration rates (Broquet et al., 2009; Martin et al., 2021), MigEst estimates gene flow based on the kinship (Wang, 2014) and therefore suitable for quantifying the contemporary and mass migration of insect species. A custom python script was used to count the number and percentage of parentage assignment in each population pair based on *.BestConfig_Ordered file generated by Colony and transform it into the input for MigEst. It is worth noting that the sex of the samples was not recorded before extracting the DNA. This might have limited effective quantification of migration among populations because the uncertainty of the paternity-offspring or maternity-offspring assignment (Jones and Wang, 2010). But as the parentage-offspring relationship was robust in Colony, the migration direction and linkage between populations remain effective and should provide useful information in investigating metapopulation dynamics (personal communication, Dr. Jinliang Wang).

### Population network analysis and pivotal node identification

Using the contemporary gene flow matrix, we performed population network analysis based on igraph (Csardi and Nepusz, 2006). We constructed two population networks based on migration matrix. The first network employed gene flow between two populations as the non-weighted directed edge and the degree centrality (Everett and Borgatti, 2005) of each site as the size of the node. We constructed the second population network by using betweenness centrality, a measure of centrality in a graph based on shortest paths (Everett and Borgatti, 2005), of each edge, and further performed the modularity clustering (Newman, 2006) based on fast greedy algorithm implemented in igraph (Csardi and Nepusz, 2006). When identifying pivotal nodes, we treated nodes that were important in population intermixing (e.g., sink populations that received immigration from different populations and mixed) or being the source of many other populations as key nodes. These key nodes generally had higher node degrees and/or being shared by two or more clusters in modularity clustering analysis.

## Results

### Genetic variation and structure

**FIGURE 2:**
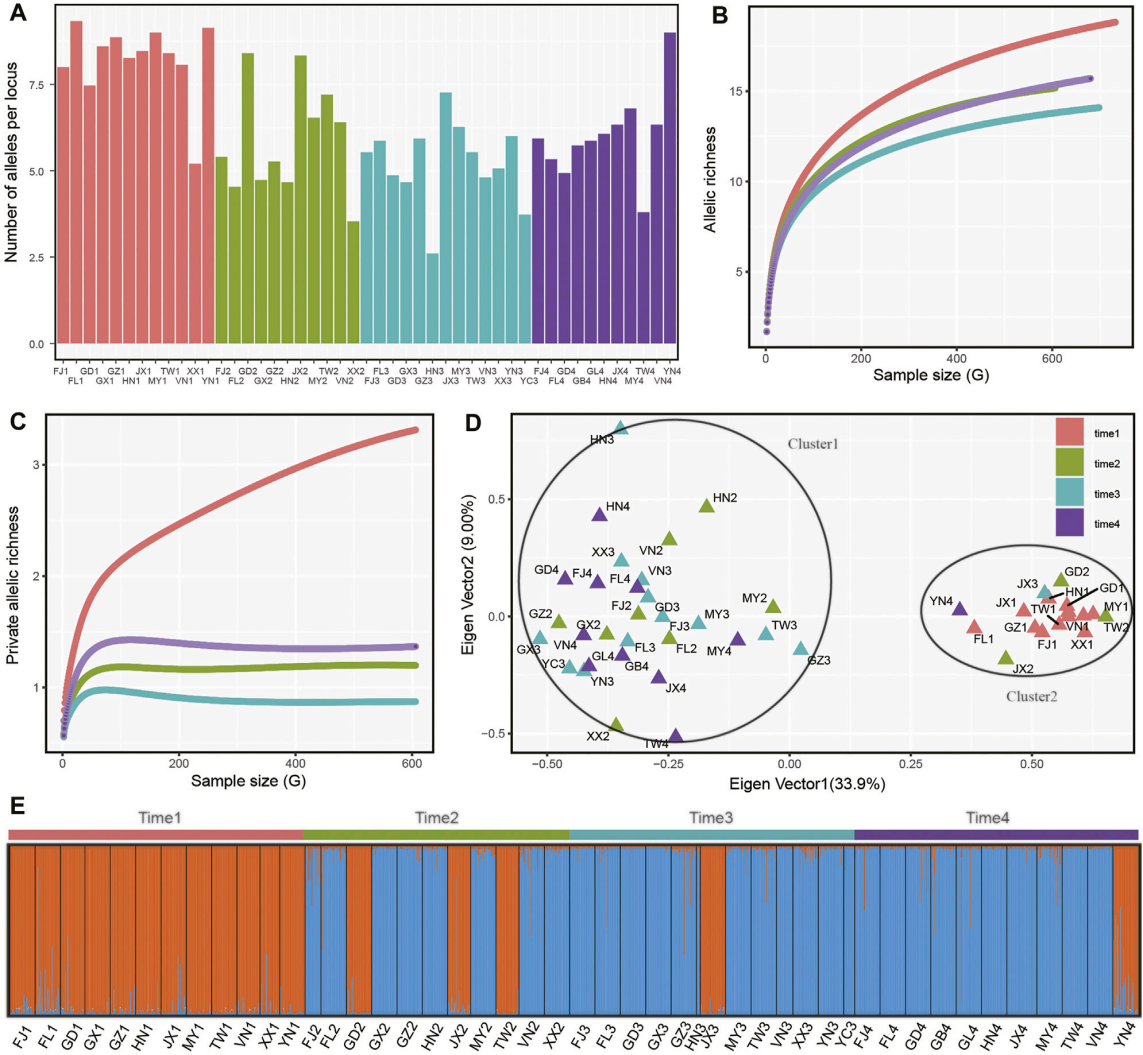
Genetic variation and differentiation of *P. xylostella* populations. **(A)** Average number of alleles per locus of each population. For other genetic parameters referred to Supplementary Table S2. **(B)** Accumulation of allelic richness in populations grouped by sampling time (i.e., a metapopulation). The sample size increased from two to the total number of alleles in each population. **(C)** Accumulation of private allelic richness in populations grouped by sampling time. **(D)** Principal coordinate analysis (PCoA) based on allele frequency of each sampling population. **(E)** Population genetic structure analysis based on STRUCTURE.

In four sampling time intervals, a total of 1,425 individuals from 48 *P. xylostella* populations were collected in South China and Southeast Asia (Table 1), with each time interval consisting of 12, 11, 13, 12 populations and 373, 334, 360, 358 individuals, respectively. Based on several metrics of genetic variation calculated in *P. xylostella* populations (Figure 2; Table 2), we found populations collected in time 1 (April–June 2016) generally had higher genetic variation than the populations from the other time intervals collected at the same or nearby site. There were some exceptions that had higher genetic variation compared with other populations collected at same sampling time interval: FJ2, TW2, and JX2 collected at time 2 (October 2016–January 2017), JX3 at time 3 (April–June 2017), and YN4 at time 4 (November 2017–January 2018) (Figure 2A; Table 2). By defining DBM populations collected from each time interval as a group (metapopulation), we further calculated mean number of distinct alleles and private alleles per locus in each group from a sampling size (i.e., G) from 2 to the maximum size of the group using iteration method. We found that time 1 had both higher mean number of distinct alleles and private alleles per locus compared with other groups (i.e., time 2 to time 4, Figures 2B,C). The saturation curve of private alleles in time 1 was not plateaued, but a clear platform was found for all other three time intervals (Figure 2C).

**TABLE 2:** Genetic diversity of *P. xylostella* populations.

Sampling time code	Pop	Na	*Ne*	*I*	*Ho*	*He*	*Ar*
time1	FJ1	8	3.558	1.437	0.546	0.654	8.43
	FL1	9.333	4.411	1.527	0.53	0.656	4.084
	GD1	7.467	3.754	1.443	0.518	0.665	4.503
	GX1	8.6	3.954	1.522	0.557	0.685	8.008
	GZ1	8.867	4.47	1.595	0.557	0.708	4.278
	HN1	8.267	4.127	1.512	0.565	0.686	4.83
	JX1	8.467	4.033	1.474	0.564	0.644	4.611
	MY1	9	4.367	1.563	0.596	0.683	8.158
	TW1	8.4	3.897	1.462	0.575	0.655	18.301
	VN1	8.067	4.129	1.501	0.579	0.681	13.174
	XX1	5.2	3.312	1.239	0.549	0.622	5.941
	YN1	9.133	4.214	1.575	0.572	0.693	9.174
	Mean	8.233	4.019	1.487	0.559	0.669	7.791
time2	FJ2	5.4	3.169	1.173	0.555	0.575	6.987
	FL2	4.533	2.913	1.084	0.459	0.567	2.747
	GD2	8.4	4.1	1.461	0.531	0.651	3.139
	GX2	4.733	2.932	1.042	0.354	0.535	5.616
	GZ2	5.267	2.62	1.038	0.362	0.508	3.478
	HN2	4.667	2.795	1.087	0.46	0.564	3.713
	JX2	8.333	3.466	1.437	0.468	0.637	4.016
	MY2	6.533	3.078	1.228	0.469	0.59	6.19
	TW2	7.2	3.797	1.426	0.515	0.655	9.368
	VN2	6.4	3.477	1.32	0.431	0.62	8.034
	XX2	3.533	1.685	0.634	0.289	0.342	4.66
	Mean	5.909	3.094	1.175	0.445	0.568	5.268
time3	FJ3	5.533	2.919	1.159	0.517	0.582	3.867
	FL3	5.867	2.855	1.183	0.471	0.583	2.192
	GD3	4.867	2.665	1.005	0.424	0.517	2.426
	GX3	4.667	2.33	0.906	0.361	0.455	2.745
	GZ3	5.933	3.243	1.28	0.471	0.625	2.358
	HN3	2.6	2.017	0.701	0.363	0.411	2.324
	JX3	7.267	3.734	1.434	0.538	0.668	2.808
	MY3	6.267	2.892	1.173	0.453	0.568	3.275
	TW3	5.533	2.852	1.181	0.488	0.6	4.291
	VN3	4.8	2.769	1.093	0.484	0.559	3.771
	XX3	5.067	2.875	1.124	0.466	0.567	2.772
	YN3	6	2.879	1.12	0.557	0.532	3.927
	YC3	3.733	2.131	0.88	0.51	0.476	3.449
	Mean	5.241	2.782	1.095	0.469	0.549	3.093
time4	FJ4	5.933	3.366	1.263	0.453	0.605	4.116
	FL4	5.333	3.179	1.242	0.446	0.631	2.45
	GD4	4.933	2.676	1.081	0.374	0.55	2.301
	GB4	5.733	3.295	1.25	0.446	0.608	3.979
	GL4	5.867	2.949	1.134	0.447	0.548	2.812
	HN4	6.067	3.017	1.148	0.438	0.554	3.005
	JX4	6.333	3.245	1.279	0.461	0.602	3.231
	JX5	2.6	1.827	0.618	0.3	0.347	4.001
	MY4	6.8	3.262	1.221	0.504	0.578	5.84
	TW4	3.8	2.149	0.892	0.351	0.49	5.201
	VN4	6.333	3.391	1.279	0.575	0.604	3.005
	YN4	9	3.738	1.478	0.525	0.65	5.134
	Mean	5.728	3.008	1.157	0.443	0.564	3.756

*Na*, *Ne*, *I*, *Ho*,*He*, and *Ar* represent number of alleles, effective population size, Shannon index, observed heterozygosity, expected heterozygosity, and allelic richness, respectively.

Further genetic structure analysis found that the populations with higher genetic variation clustered together (cluster1), while the other populations formed a second cluster (Figure 2B). We double checked the genotyping data and found that this pattern in our study was not due to genotyping error of SSR makers but due to coexistence of two DBM swarms. Populations from cluster1 were mainly collected from time 1, while some populations in this cluster also being found in isolated regions (i.e., Guangdong, Jiangxi, Taiwan and Yunan in China) collected in the subsequent sampling times (Figures 2D,E). Similarly, the genetic structure analysis with the best K value of 2 showed that cluster1 was the dominant insect swarm in time 1, while only three populations from cluster1 were found in time 2 (GD2, JX2, TW2). In time 3 (JX3) and time 4 (YN4), only one population from cluster1 was identified. Within each sampling time, we further performed correlation analysis based on Nei’s genetic distance and geographical distance of each population pair and found no significant correlation in the four datasets (time 1: *r* = 0.063, *p* = 0.808; time 2: r = −0.133, *p* = 0.502; time 3: r = −0.026, *p* = 0.889; time 4: r = −0.037, *p* = 0.853).

### Source-sink populations and migration distance

The parentage analysis based on Colony identified the best configuration of offspring and parental individuals in each sampling time interval and was used to infer contemporary gene flow between source-sink populations. We found similar migration events among populations identified by markers using different error rates. This supports the accuracy of kinship assignment analysis (Wang, 2018). We thus used the estimation with an error rate of 0.0001 for further analysis.

**FIGURE 3:**
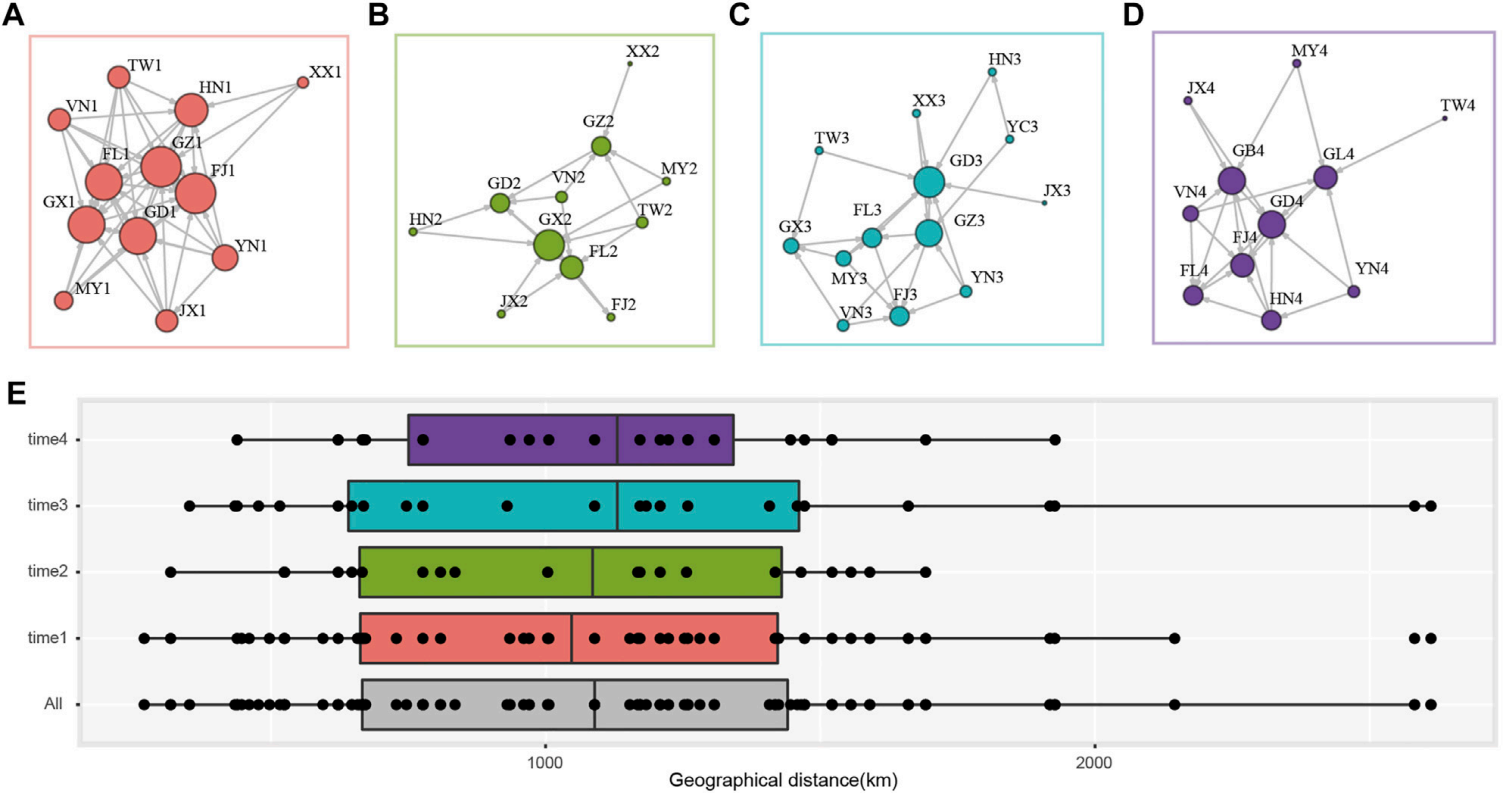
Gene flow and migration distance of sampling *P. xylostella* populations. Migration network of each sampling time. **(A)** time1; **(B)** time2; **(C)** time3; **(D)** time4. The presence of gene flow between populations was indicated with directed and non-weighted edge, and the degree centrality was represented by the size of each node. **(E)** The geographical distance (km) of population pairs with gene flow. time 1 to time 4 indicate population pair in each time interval, and “All” denotes all the population pairs. Boxes show the first and third quartile range (IQR) in each panel.

The population network was constructed using the presence of gene flow between two populations as edge, with the arrow representing the direction and the size of node indicated the degree centrality (i.e., number of the connected nodes). In time 1, FJ1, FL1, GD1, GX1, GZ1, HN1, and JX1 were with high degree centrality and receiving immigrants from other populations (Figure 3A), while FJ2, FL2, GD2, GX2, and GZ2 in time 2 were sink populations and GX2 has the most connected populations (Figure 3B). In time 3 and time 4, FJ3, FL3, GD3, GX3, GZ3, HN3, and FJ4, FL4, GD4, GB4, GL4, HN4 received immigrants (Figures 3C,D). We found a pattern where DBM populations from Fujian (FJ), the Philippines (FL), Guangdong (GD, GB), Guangxi (GX), Guizhou (GZ) always received contemporary immigration and were the sink populations irrespective of the sampling time and wind direction. While regions such as Yunnan (YN) and Taiwan (TW), Vietnam (VN), and Myanmar (MY) located at peripheral regions and encircled the sink populations, they were always the source populations in four sampling times (Figure 3). Several populations [e.g., Hainan (HN)] were the source and sink that varied across sampling times.

We further summarized the geographical distance of each population pair with gene flow. A total of 115 population pairs with contemporary gene flow in the overall 249 pairs were identified (Figure 3). The data showed that DBM could migrate more than 2,500 km although there were only few successful immigration events at this distance (Figure 3E). Most of the migration events occurred at around 1,000 km, with a 95% confidence interval of 1,003.45 km–1,196.77 km. We found similar migration distance in each time interval, but only in time 1 and time 3 (during the spring of each year), we found migration events over 2,500 km (Figure 3E).

### Migration network clustering and key nodes for temporal metapopulation dynamics

**FIGURE 4:**
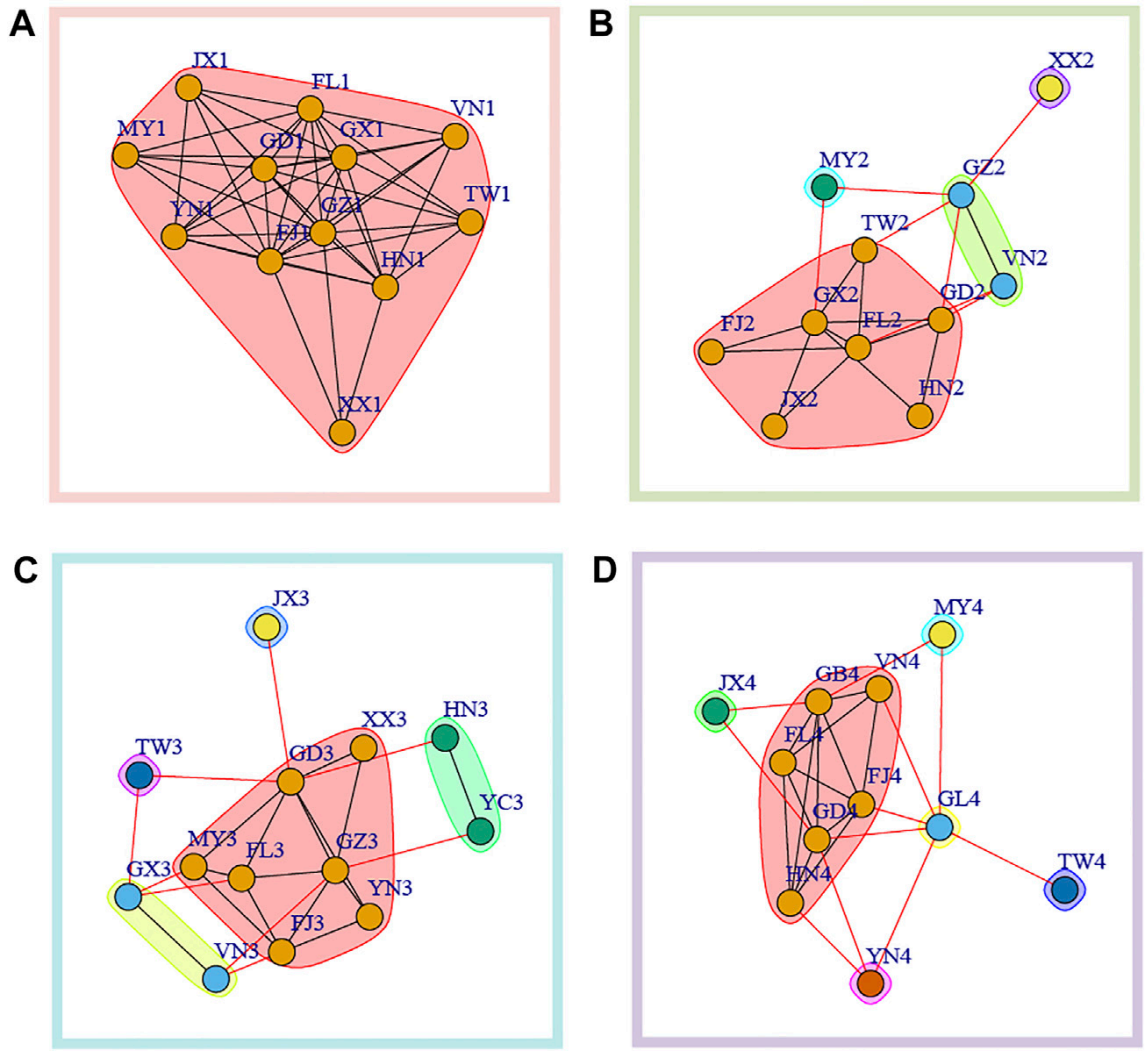
Modularity clustering analysis based on betweenness between populations in each sampling time interval. **(A)** time1; **(B)** time2; **(C)** time3; **(D)** time4.

We further used Newman’s modularity clustering in igraph to construct the migration network. To distinguish from the former network, we used betweenness as undirected and weighted edges. Overall, we found a highly differentiated pattern of clustering across sampling time intervals, and clustering analysis found samples from spring (time 1 and time 3) have lower clustering number compared with that in winter (time 2 and time 4) (Figure 4). At time 1, all the populations formed one cluster (Figure 4A). At time 2, four clusters were identified, with Myanmar (MY2) and Hunan (XX2) each forming independent clusters. The third cluster consisted of Guizhou (GZ2) and Vietnam (VN2) while the rest populations formed the fourth cluster (Figure 4B). At time 3, five clusters without overlapping were identified. At time 4, we further found six clusters with Jiangxi (JX4), Guizhou (GL4), Yunnan (YN4), Taiwan (TW4) and Myanmar (MY4) forming individual independent clusters.

Combining network clustering (Figure 4) and centrality (Figure 3), we were able to identify some key nodes that could be important in metapopulation dynamics. Basically, we considered the populations with higher centrality were key nodes serving as the sink for genetic intermixing (mixing of standing variation and/or *de novo* mutations) or as source population that could mediated gene flow into the other populations. In time 1, highest degree centrality was found in Fujian (FJ1) and Guizhou (GZ1). Across four clusters of time 2, Guangxi (GX2) was the population with the highest node degree (Figure 4B). Of five clusters at time3, we found that Guangdong (GD3) had the highest degree centrality (Figure 4C). In time4, Guangdong (GD4) and Guizhou (GB4) within the same cluster in the modularity clustering analysis had highest degree centrality (Figure 4D).

## Discussion

### Windborne migration mediates rapid population turnover in overwintering regions

For many insect pests, resources and habitats are ephemeral across time and space (Osborne and Woiwod, 2002) due to fast turnover of modern agriculture. Windborne migration of agricultural pests is important in escaping unfavorable habitats and sustaining succession of insect populations. In the sampling region, we found two *P. xylostella* swarms across all sampling populations, with one replaced by the other over time. This result suggested that windborne migration of this pest has facilitated a rapid population turnover across the metapopulation of *P. xylostella* in South China and Southeast Asia. Similar pattern of replacement has also been identified in other studies of insect pests. For example, Dinsdale et al. (2011) report rapid population turnover of *Bemisia tabaci* over time when investigating the impact of agricultural landscapes on its population genetic pattern. Working on temporal variation in genetic composition of *Helicoverpa zea* (Boddie), Perera et al. (2020) find that the pest populations tend to differentiate over time, but suggest that this can be a transient or temporary phenomenon. We thus proposed that the rapid population turnover of DBM should be taken into consideration for effective pest management such as temporal insecticide resistance monitoring under modern agricultural regime, as it might be for other species.

### Contemporary population network clustering and key nodes for source-sink dynamics

Based on modularity clustering analysis, we found a differentiated clustering pattern across different sampling times, which supported a dynamic metapopulation of *P. xylostella* in overwintering regions. This could be explained by low level of autonomous control over migration direction (Gao et al., 2020) and the variation of regional wind directions and strength (Coulson et al., 2002). We found most of the migration distances between populations were about 1,000 km, which were similar to previous analysis based on genome-wide SNPs (Chen et al., 2021). The relatively “short” migration distance over large geographical scale of *P. xylostella* metapopulation in China not only support step-stone migration (Chen et al., 2021) that forms migration circuits of this pest, but could also contribute to temporal population clustering, likely regulated by seasonal winds. For example, we find a differentiated pattern of population clustering between seasons in two consecutive years (i.e., time 1 vs*.* time 2, and time 3 vs. time 4). To be specific, less clusters in spring (time 1 and time 3) compared with that in winter of the same year (time 2 and time 4) were observed. This pattern is likely due to more active windborne migration in spring that contributed to the linkage across regional populations (Fu et al., 2014). More active migration in the spring was also evidenced by migration events over 2,500 km were only identified in time 1 and time 3, while not in winter populations (time 2 and time 3). A combination of strain-specific characteristics with regional wind strength and directions (Coulson et al., 2002) as well as particular “weather window” thus could explain the temporal variation of population network and dynamics of this pest. Further research (e.g., energy reserve of *P. xylostella* individuals) should be conducted for a comprehensive understanding of migration at certain geographical distances and its contribution to regional metapopulation dynamics.

In addition, we found that populations from Southwest of China (e.g., Yunnan) as well as Indo-China Peninsula (ICP, e.g., Myanmar and Vietnam) were always the source for populations in our four samplings. This pattern has also been identified in a recent analysis based on genome-wide SNPs (Chen et al., 2021), where populations of Yunnan are the source in two consecutive years. While the populations from ICP were not sampled in Chen et al. (2021)’s study, previous analysis of many insect pests have found this region to be the source of insect immigration in China based on trajectory modeling [e.g., *Nilaparvata lugens* (Stål) (Hu et al., 2014); *Sogatella furcifera* (Sun et al., 2017); *Spodoptera frugiperda* (Li et al., 2020)] and genetic analysis [e.g., *Nilaparvata lugens* (Stål) (Hu et al., 2019)]. The year-round suitable temperature in Yunnan and Indo-China Peninsula (e.g., northern and central Vietnam, Laos, and northeastern Thailand) may be the reason for maintaining a large number of populations ready for migration. We thus suggest that these regions should not only be important for effective control of rice pests (Hu et al., 2014; Sun et al., 2017), but also for the management of other important vegetable pests, such as *P. xylostella*. We additionally found several regions such as Fujian and Guangdong were always being the receivers in these regions, a pattern that was consistent over 2 years. These regions may be in the migration corridor for *P. xylostella*’s migration, similar to Beihuang Island in Bohai gulf (Fu et al., 2014). Nevertheless, this pattern should be verified by more dense sampling of populations from both geographical and temporal scales as it was contradicted our the general understanding of monsoon circulation in East Asia (Hu et al., 2019).

### Incorporation of contemporary gene flow and population network in pest management

Current insect ecology has witnessed a boom in population genetics/genomics studies of agricultural pests [www.mdpi.com/journal/insects/special_issues/population_genetics, e.g., Sethuraman et al. (2020)]. Research has advanced our understanding of insect genetic variation and differentiation under globalization. In insect ecology and pest management, choosing the proper scale and right parameters is important for understanding population dynamics and connectivity (Osborne and Woiwod, 2002). Genetic parameters, such as *N*_m_, that represent historical gene flow are unsuitable for partially migrating populations of agricultural insects (Menz et al., 2019). Pedigree-based approaches that identify instant migration events should be a better choice (Martin et al., 2021).

Investigation of contemporary gene flow is important especially for insecticide resistant pest monitoring. For example, Wang et al. (2022) report higher frequency of insecticide resistant alleles in *P. xylostella* populations crossing the Bohai Strait during the spring compared with those in winter. This temporal differentiation of insecticide resistance strains is likely due to variation in insecticide pressure between Southern and Northern China (Wang et al., 2022). Further studies incorporating both neutral markers and selective loci should benefit disentangling factors (e.g. migration or adaptation) contributing to the formation of insecticide resistance. Nevertheless, populations that were consistent source populations, which were identified from population network, should be monitored for their insecticide resistance throughout the year to help define plans for proactive pest management. For sink populations to where insecticide resistant insects migrate, application of the spatially common chemicals should be delayed or avoided to reduce the potential of insecticide-resistance development. In addition, crop rotation such as cultivating non-brassicaceous vegetables (Li et al., 2016; Ke et al., 2019) could be used to reduce source-sink connection among regions. Contemporary dynamics and population network analysis could be used to identify source-sink connections and regional metapopulation dynamics and thus should benefit effective management of highly dispersive insect pests.

## Conclusion

In this research, we investigated the temporal variation in genetic makeup of *P. xylostella* across its overwintering region in Southern China and Southeast Asia. Our results depicted a rapid population turnover and dynamic metapopulation of this highly dispersive pest and highlighted the importance of temporal sampling and population network analysis in understanding contemporary genetic pattern and regional source-sink dynamics of agricultural insect pest. These results are expected to shed light on monitoring of the real-time dynamics of insecticide resistance and identify regional key populations towards more effective and sustainable management in the long term. More broadly, methods of this study could also be applied to other highly dispersive insect pests under modern agricultural regime.

## Data Availability

The datasets presented in this study can be found in online repositories. The names of the repository/repositories and accession number(s) can be found in the article/Supplementary Material.
